# ICD-11: A catalyst for advancing patient safety surveillance globally

**DOI:** 10.1186/s12911-023-02134-2

**Published:** 2023-03-09

**Authors:** Alan J. Forster, Christopher G. Chute, Harold Alan Pincus, William A. Ghali

**Affiliations:** 1 The Ottawa Hospital Ottawa; Ottawa Hospital Research Institute, Clinical Epidemiology Program; and Faculty of Medicine, University of Ottawa, Ottawa, Canada; 2 Schools of Medicine, Public Health, and Nursing, Johns Hopkins University, Baltimore, USA; 3 Irving Institute for Clinical and Translational Research and Department of Psychiatry, Columbia University and New York State Psychiatric Institute, New York, NY USA; 4 Office of the Vice President Research; and, The O’Brien Institute of Public Health, University of Calgary, Calgary, AB Canada

**Keywords:** Patient safety, Surveillance, International classification of diseases, ICD-11

## Abstract

The World Health Organization’s (WHO) international classification of disease version 11 (ICD-11) contains several features which enable improved classification of patient safety events. We have identified three suggestions to facilitate adoption of ICD-11 from the patient safety perspective. One, health system leaders at national, regional, and local levels should incorporate ICD-11 into all approaches to monitor patient safety. This will allow them to take advantage of the innovative patient safety classification methods embedded in ICD-11 to overcome several limitations related to existing patient safety surveillance methods. Two, application developers should incorporate ICD-11 into software solutions. This will accelerate adoption and utility of software-enabled clinical and administrative workflows relevant to patient safety management. This is enabled as a result of the ICD-11 application programming interface (or API) developed by the WHO. Third, health system leaders should adopt the ICD-11 using a continuous improvement framework. This will help leaders at national, regional and local levels to take advantage of specific existing initiatives which will be strengthened by ICD-11, including peer review comparisons, clinician engagement, and alignment of front-line safety efforts with post marketing surveillance of medical technologies. While the investment to adopt ICD-11 will be considerable, these will be offset by reducing the ongoing costs related to a lack of accurate routine information.

## Introduction

More than 20 years after the US National Academy of Medicine published “To Err Is Human”, health systems are unable to confidently and unequivocally state patient safety has improved [1]. While there are many reasons for this, the root cause is a continued inability to consistently measure adverse events and potential adverse events. Providers, hospitals, payers, and governments continue to rely on different measurement approaches lacking sensitivity, specificity, and reliability [2]. As a result, improvement efforts are often unsuccessful and the oft quoted maxim ‘You can’t manage what you can’t measure’ continues to hold true for patient safety. While measuring patient safety will not result in improvement, efforts to improve can only be judged beneficial if there are reliable measurements.

Adoption of the International Classification of Disease version 11 (ICD-11) offers an opportunity to address this gap. When the World Health Organization (WHO) developed earlier versions of the ICD, the measurement of patient safety was less of a priority. Consequently, the ICD did not include a comprehensive and consistent approach for documenting patient safety events, and as a result, the use of ICD codes fell short of the needs of those responsible for tracking patient safety.

Unlike prior versions, the ICD-11 was built with patient safety measurement in mind. The WHO assembled an international group of experts and tasked them with ensuring the ICD-11 met this use case. The so-called Quality and Safety Topic Advisory Group created the coding rules and reviewed the code content to ensure all safety events could be tracked. The group incorporated leading patient safety nomenclatures and evidence to establish its three-part model, which tracks the harm, the cause, and the mode for every event (as described in another article in this series). The ICD-11 also includes new features such as clustering of diagnosis codes, diagnosis timing, and extension codes that enhance capture of clinical information for quality and safety. This creates a classification that is more flexible, intuitive, and clinically aligned—ensuring it can be consistently applied to several purposes and in diverse settings [3].

In order to take advantage of the opportunities afforded by ICD-11, national and/or regional health systems will need to manage the adoption of the new coding system. A full implementation will take some time; however, it is unnecessary to wait until full deployment for benefits to be accrued, especially if certain issues are addressed. First, it is necessary for leaders (within health systems and at system/national levels) to consistently apply the ICD-11 classification system across all approaches for monitoring patient safety. Second, the adoption of ICD-11 by manufacturers of health information systems and electronic medical record components could accelerate change. Third, leaders need to use the information generated from adoption of ICD-11 to guide system improvement. We address each of these in turn.

## Suggestion 1: Incorporate ICD-11 into all approaches to monitor patient safety

There are four basic approaches to monitor patient safety. These include voluntary reporting of safety events, mining healthcare administrative data, mining clinical data, and prospectively observing care for safety events [4]. As described in Table 1, each method has specific benefits and drawbacks. Currently, the ICD is predominantly used only for mining healthcare administrative data. While using ICD for mining administrative data is beneficial, ICD-11 creates an opportunity to extend a single classification system to each of the four approaches mentioned above, as ICD-11 was designed to incorporate conceptual elements from pre-existing taxonomies, such as the WHO’s International Classification for Patient Safety (the key elements of which are now embedded within ICD-11) and the Agency for Healthcare Research and Quality’s Common Formats [3].

**Table 1:** Patient safety measurement approaches

Method	Description	Benefit	Drawback	ICD-11 based improvements
Voluntary reporting	Healthcare workers report events when they occur using standard forms	Inexpensive, engages staff in safety culture, can capture emerging risks	Reporting behavior drives event capture	Improved communication of what to report; consistency with other approaches
Mining administrative data	Hospital claims data including ICD codes are used to generate “Patient Safety Indicators”	Inexpensive, ability to aggregate measures at hospital, jurisdiction, and national levels	Code capture is influenced by several factors. Codes are not mutually exclusive nor completely exhaustive	Code capture will be improved; codes are now mutually exclusive and completely exhaustive
Mining clinical data	Electronic medical record data are used to generate “Patient Safety Indicators”	Inexpensive, clinically relevant, flexible	Depends on availability of data; lack of consistency between manufacturers; not built-in	Alignment of indicators with higher order concepts
Prospective observations	Observers track patients along their illness trajectory to identify prespecified events	Clinically relevant and acceptable, adaptable to many environments, does not require information technology	Expensive	Consistency with other approaches, enabling development of machine learning and other AI based approaches to detection

The current state (prior to ICD-11) leaves us with a lack of interoperability across different measurement approaches. This is because there is no single terminology applicable to all safety measurement approaches. Therefore, it is essentially impossible to compare events across the measurement approaches as different terminologies are used to define and group concepts. Differences in approaches lead to variation in measures that are unacceptable to most users. By applying the same framework inherent to ICD-11 to all the use cases, this problem immediately goes away.

For example, if a harm associated with a long-used medical device becomes apparent today it is very difficult (and perhaps) impossible to scan results from all the different approaches to identify any signals that could help to estimate the overall risk. This is because all four methods for tracking patient safety use different nomenclatures to describes the events. ICD-11 on the other hand makes this relatively straightforward because a single nomenclature and coding rules can be applied for all four methods to track safety [5]. If all the four methods employed all components of the ICD-11 including the three-part model (described elsewhere in this article series), then this type of analysis would be greatly facilitated. By creating an ability for patient safety leaders to compare information arising from all four methods using a common language, they will be in a better position to select priorities and monitor impact of interventions.

A second limitation of the existing situation is a failure to adopt standards even within a measurement approach. For example, voluntary reporting systems typically have their own proprietary embedded classification that differ from one another [6]. Similarly, electronic medical records do not routinely map safety concepts to an accepted standard [3]. Thus, comparing results across institutions with different systems becomes impossible. Further, if developers in an institution code a set of electronic triggers indicating events, then it is likely their results will not be comparable to other institutions. Prospective observations also share this limitation; however, they are all obviated by consistent implementation of ICD-11.

A third limitation of the existing situation is scalability. Even if existing systems can capture basic components of a patient safety event there is an inability to grow them. For example, within existing classification systems, when new pharmaceutical products or medical devices become available, then it is necessary to update the entire coding system to accommodate additions of the new products and their relationships to other concepts.

The ICD-11 exists within the WHO’s Family of International Classifications [7]. This introduces an opportunity to integrate the International Classification of Health Interventions, the International Classification for Functioning and Disability, and controlled terminologies for medications and devices embedded with the extension code section of ICD-11. These other classifications systems can evolve independently while maintaining appropriate relationships within the ICD-11 and importantly the three-part patient safety framework.

A transition to these three WHO systems is certainly a tall order for any country but it is the type of large-scale change that could transform health information systems. Using standard nomenclature within these core concepts will allow for more accurate measurement, which in turn will support more effective improvement actions [1]. Moving forward, the classification systems and code sets related to these embedded concepts can change and evolve without impacting ICD-11 while remaining relevant to the patient safety use case.

## Suggestion 2: Incorporate ICD-11 into software solutions

**Fig. 1:**
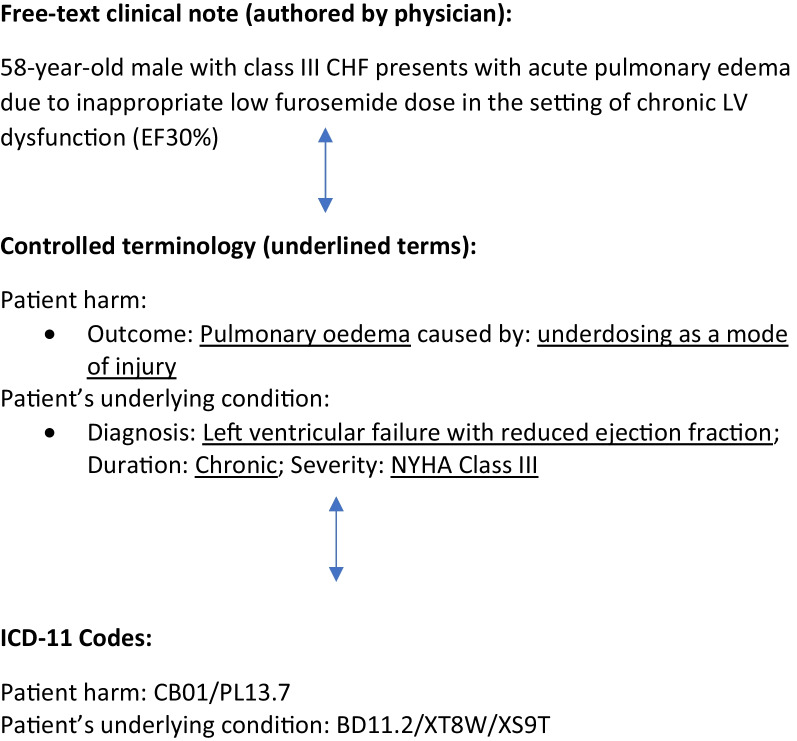
Example of ICD-11 terms and codes derived from a clinical note

**Fig. 2:**
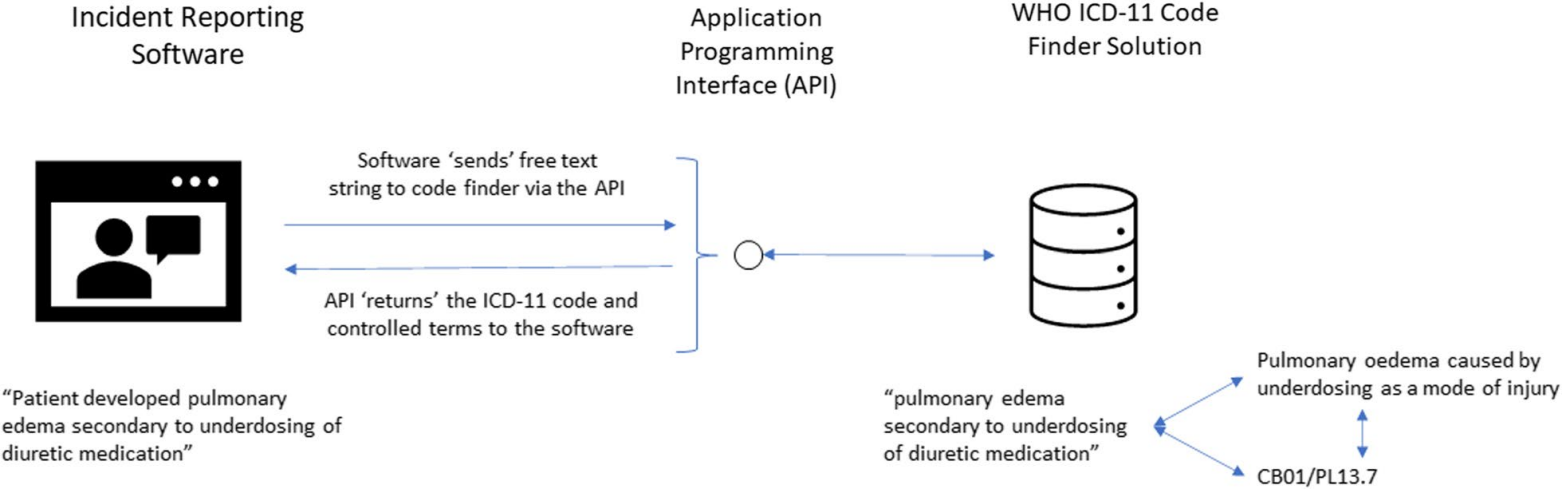
Application programming interface facilitates patient safety workflow by linking software solutions (for example incident reporting software) and the WHO ICD-11 code finder solution

To achieve success, it is important to build the ICD-11 into solutions facilitating the workflow of busy patients, clinicians, and health system leaders [8]. Specifically, we recommend ICD-11 be built into solutions related to incident reporting, clinical documentation, performance reporting, and automated artificial intelligence-based approaches for safety event detection. Physicians and hospital staff document care using natural language or free text, not using controlled terminology or alphanumeric codes. Thus, most health systems must employ staff to manually translate free text within records into the ICD codes, which are then used for a number of purposes. This process is inefficient and can lead to errors in code assignment [9]. Now that the WHO has created code finder software and an associated Application Programming Interface (or API), there are now relatively straightforward approaches for software developers to automatically link free text within their applications to controlled clinical terminologies (i.e., the terms embedded within the foundation layer of ICD-11, or proprietary terminologies such as SNOMED) and then to ICD-11 codes (see Figs. 1 and 2) [10, 11]. This capability means that we can theoretically obtain globally consistent coding irrespective of the language or system used to document care, if developers use the WHO Code Finder software and API. Of course, there remains a need to confirm the accuracy of code assignment. However, with increasing numbers of software solutions created using the API, we predict coding accuracy will also increase.

Incident reporting is a cornerstone of patient safety management. One limitation of existing applications is the inconsistent classification across voluntary reporting systems, which limits comparisons between institutions using different applications [6]. Another limitation is the limited clinical acceptability of existing classification systems [6]. The terminology used in voluntary reporting systems do not match clinical terminologies [6]. This makes the information contained within the incident reporting systems cumbersome to use and limits uptake by healthcare workers. Furthermore, the existing systems do not lend themselves to patient-reported outcomes. By using clinically relevant terms, it is easier for patients to report into the systems. This might greatly enhance the utility of incident reporting systems, and in turn, patient safety culture, which is thought to be a prerequisite to improving safety [6].

A second domain for potential improvement is within clinical documentation systems themselves. Historically, clinical documentation has been based on free text. This is a legacy of written notes. With electronic medical records, there remains a free text component, but it is now possible to create required fields with structured responses. If done correctly, it becomes possible to structure the documentation within the medical record to include appropriate information for each safety event. For example, for a medication-induced rash, a structured response could include a) documentation of the type and extent of the rash (e.g., maculopapular rash on trunk and extremities), b) the causative agent (e.g., amoxicillin), and c) whether the therapy was appropriately prescribed and administered (e.g., correct dose and route and no history of a drug allergy). By requiring documentation in a standard manner, the electronic record will provide much higher quality information. Importantly, systems need to be built to consider clinician workflow.

A third domain of application development is within performance reporting systems that extend beyond individual hospitals. Frequently, electronic medical record systems are built at the clinic, nursing unit, and hospital level without thinking of the higher-level system goals. As a result, clinical workflows for individual patients are considered, without incorporating population health management needs [12]. This limits ability to track event rates at a population level because it is difficult to determine the true at-risk population. One way to address this is to define the performance reporting needs at the population level and build automated reports to present the information. From this higher-level perspective that considers system needs, developers can then map the required data elements.

For example, if tracking medical device related harms (e.g., implanted pacemakers), it is likely that system managers will want to track utilization of devices (e.g., by type/manufacturer), the proportion of utilization resulting in harm (and type of harm), the severity of harm resulting from the device, and the mechanisms by which harm occurs for a particular device (e.g., battery problems). Further, the manager will want to link information on harms with information on the patients affected, the providers involved, and the settings in which the device was used. Rich details describing the harms within a particular device will allow the manager to be more precise in action to improve safety. By anticipating the report content, developers can develop reports that will take advantage of the rich information contained within electronic records. This benefit becomes extremely meaningful because of the ability to work across electronic systems and safety measurement approaches.

A final domain of development is related to event detection using artificial intelligence. To enable accurate detection, it is necessary to have consistent outcomes [13]. For example, renal dysfunction following prescription of an angiotensin converting enzyme inhibitor. The application of machine learning to patient safety detection will be greatly improved, as consensus grows around agreed upon outcomes (e.g., the extent of renal impairment considered minimally clinically relevant) that need to be evaluated across systems [13]. Thus, if an algorithm can detect outcomes in one system, then the same algorithm can be calibrated in other systems. Without a consistent classification of outcomes across systems, this would not be possible.

## Suggestion 3: Health system leaders adopt the ICD-11 using a continuous improvement framework

There are several opportunities to improve health system management with respect to patient safety. It is important, however, for health leaders to recognize where adoption of ICD-11 will reinforce their efforts and to use these opportunities as a burning platform upon which to stimulate change. These areas might include the following—engagement of clinicians in safety, improved peer comparison, and alignment of efforts between grass roots quality improvement teams and health technology regulators [14].

A significant downstream impact of measurement challenges is consistent buy-in from clinicians and other stakeholders [15]. If measures are non-sensical, then clinicians will not find the results compelling enough to change their behavior, especially if the desired change is less efficient. Thus, a consistent measurement approach that is clinically relevant and consistent across measurement approaches will be a powerful catalyst for engaging local professionals to work together on common problems (and directly link this work to similar efforts at national and even global levels).

For example, if the administrative data suggest that surgical site infections are a problem, then several investigations could be launched to investigate. The incident reporting system can be interrogated to determine the cases with infection, a prospective surveillance program could be launched to identify infections going forward, and the EMR could be interrogated to investigate the association of case duration, intra-operative temperature loss, and compliance with perioperative antibiotics with the risk of outcome. ICD-11 creates a consistent approach for outcome determination, making it much more likely to get clinicians involved in believing the results of the administrative data and to participate in such reviews.

The second area where leaders need better information is related to comparisons [15]. The lack of an accurate and reliable measurement system results in expensive efforts to improve and an inability to judge the impact. For example, in order to set priorities, it is often necessary to judge relative performance—either against peers or over time. If a provider has a higher adverse outcome rate than peers or their outcome rate is getting worse over time, then it makes sense to set actions to prevent the adverse outcome as a priority. This type of logic only works if the information guiding the priority setting is valid [16]. The more structured ICD-11 content in patient safety has the potential to yield greater confidence in measurements.

The third opportunity is to align the efforts of grass roots clinical teams and system leaders responsible for post-marketing surveillance of regulated products. Currently, there are a variety of approaches and ontologies are being used at national levels and, consequently, there are inconsistent approaches to aligning terminologies and concepts [17]. The ICD-11 can facilitate post-marketing surveillance by making it possible to more easily search for outcomes related to the use of products or looking for any outcomes related to products. Upon establishing a common classification system for use across all safety measurement systems, the ability to perform surveillance then increases many-fold.

There is a need for caution with these suggestions. For example, there are several very effective initiatives at national levels to engage patients and providers in patient safety activities. These include the FDA’s Adverse Event Reporting System, the Vaccine Adverse Event Reporting System and the Manufacture and User Facility Device Experience Reporting System, amongst others. If leaders accountable for these systems changed the user interface, then the people using the system may experience challenges using the reporting system, which may diminish their willingness to report. One way to lessen this potential negative impact is to leave the user interface unchanged (i.e. how reporters submit events) while mapping the reported events to the ICD-11 coding system. This would allow the user experience to remain unchanged but would enable data to be compared with other data systems as described.

Furthermore, while these opportunities are compelling for change, it is important to consider cost. From our perspective, the cost of moving to standardized information systems and frameworks, while not inconsequential, is likely be dwarfed by the massive unseen cost of continuing with the status quo. Table 1 outlines the impact of the status quo on patient safety measurement and the benefits of implementing ICD-11 for that measurement system. There is a cost of maintaining the existing non-standard approach to measurement, including the cost of reconciling the different sources of information. This is non-trivial and may alone cost more than converting to a new standard approach. Second, the management costs of engaging clinicians to work on something is extraordinary. Third, money can be spent on improvement activities of uncertain significance. Thus, leaders may divert resources to something relatively unimportant when there are critical issues that remain unaddressed.

## Discussion and conclusion

An important question for health leaders is whether patient safety is truly at the foundation of their organizations’ efforts to improve healthcare quality? If their answer is yes, then the adoption of ICD-11 must be considered as a key early step toward organizational transformation. The adoption of ICD-11 will establish a foundation for defining patient safety events. Upon this foundation, it becomes possible to develop a consistent measurement approach derived from innovative electronic documentation systems, thus enabling strategic approaches to improving system performance. In short, a failure to implement ICD-11 will likely ensure that in another twenty years after the publication of ‘To err is human’, we will continue to be asking ourselves whether patient safety has truly improved.

The clinical note consists of free-text documentation by a clinician, meaning the clinician was ‘free’ to document whatever they please in the manner of their choosing. This results in terms being used which can be imprecise and inconsistently applied, in turn making it difficult to use by a computer. The controlled terminology consists of ‘approved’ words and phrases to describe the intended concepts within the clinical note. These terms are precise and consistent, which enables a machine to unambiguously use them for processing. The ICD-11 codes are groups of alpha-numeric characters assigned to concepts by the WHO. These codes are precise and consistent and allow for the creation of relationships between codes which simplifies their incorporation in coding applications. Concepts described by groups of codes are linked using the ‘forward slash’. For example, the patient harm event has been reconciled to two codes (CB01 for pulmonary edema and PL13.7 for underdosing as a mode of injury’ linked by the forward slash. The WHO has published and made available an Application Programing Interface or API to support the incorporation of the WHO’s code finder software (which automatically links free text to terms and codes) into other applications, such as electronic medical records. This API will enable supported workflows in which free text clinical notes can be automatically translated into controlled terms and codes, which can be verified for accuracy.

A clinical user enters the incident using the user interface embedded in the reporting software. The API enables interaction between the incident reporting software and the WHO ICD-11 Code Finder Solution. This application identifies the terms and codes associated with the free text. The API provides those data to the reporting software. The user can use this information to refine or confirm what they have entered and to support statistical analysis and reporting. This capability is very powerful because it automatically solves the problems related to different users employing different free text with the same meaning.

## Data Availability

Not applicable. This is a commentary article and did not involve the collection of data

## Abbreviations

ICD: International classification of diseases
ICD-11: International classification of diseases, 11th revision
SNOMED: Systematized nomenclature of medicine
WHO: World Health Organization
